# Long-Term Follow-Up of Peripheral Pigmentary Retinopathy in Asian Patients with Danon Disease

**DOI:** 10.3390/genes11111356

**Published:** 2020-11-16

**Authors:** Jee Myung Yang, Beom Hee Lee, Gi-Byoung Nam, June-Gone Kim, Joo Yong Lee

**Affiliations:** 1 Department of Ophthalmology, Asan Medical Center, University of Ulsan College of Medicine, 88, Olympic-Ro 43 Gil, Songpa-gu, Seoul 05505, Korea; jeemang87@gmail.com (J.M.Y.); junekim@amc.seoul.kr (J.-G.K.); 2 Medical Genetics Center, Asan Medical Center Children’s Hospital, University of Ulsan College of Medicine, 88, Olympic-Ro 43 Gil, Songpa-gu, Seoul 05505, Korea; mdlbh@hanmail.net; 3 Department of Cardiology, Asan Medical Center, University of Ulsan College of Medicine, 88, Olympic-Ro 43 Gil, Songpa-gu, Seoul 05505, Korea; gbnam@amc.seoul.kr

**Keywords:** Danon disease, Asian, pigmentary retinopathy, peripheral retinopathy, inherited retinal disorder

## Abstract

Background: Peripheral pigmentary changes are common amongst women with Danon disease; however, there is currently a lack of longitudinal observational studies of the retinal changes in this condition, and the long-term visual prognosis is not well understood. Methods and Results: In this report, we present long-term follow-up data (12 years of follow-up) regarding peripheral retinopathy in an Asian woman and her mother who were both diagnosed with Danon disease. Both patients showed a novel nonsense mutation of the *LAMP2* gene (c.123 of exon 2). During the follow-up period, no evident extension of peripheral pigmented lesions or visual field progression was observed. Conclusions: We report, for the first time, the long-term longitudinal follow-up of Danon disease-related retinopathy in an Asian patient featuring an indolent macular-sparing peripheral lesion.

## 1. Introduction

Danon disease (OMIM #300257) is a rare X-linked dominant cardioskeletal myopathy disorder with multi-systemic manifestations, caused by mutations in the lysosome-associated membrane protein 2 (*LAMP2*) gene [1]. Visual and retinal pathology are observed in 69% of patients; peripheral pigmentary retinopathy is common among female patients [2,3]. However, there are no longitudinal observational studies on peripheral retinopathy in Danon disease, and the long-term prognosis (>10 years) is not well understood [3]. Here, we present the first longitudinal report of indolent peripheral pigmentary retinopathy in Danon disease and describe disease progression in an Asian woman and her mother with a previously unreported *LAMP2* mutation.

## 2. Case Report

**Figure 1:**
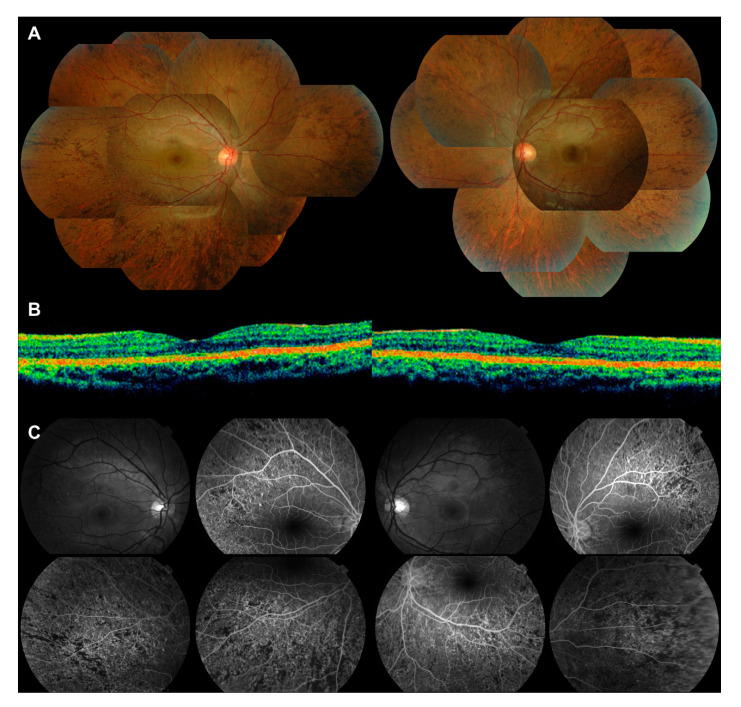
Peripheral retinopathy associated with Danon disease in a female Asian patient. (**A**) Images taken at initial presentation (21 years old). Mosaic fundus photographs of both eyes show peripheral pigmentary retinopathy with a diffuse salt-and-pepper pattern of lesions (top); (**B**) optical coherence tomography (OCT) images of the macula reveal no abnormalities (bottom); (**C**) fluorescein angiography images of both eyes show diffuse retinal pigment epithelial atrophy confined to the peripheral retina.

**Figure 2:**
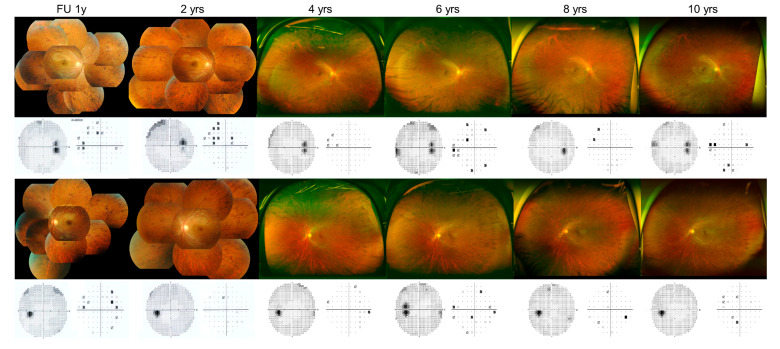
Serial peripheral fundus images and the visual fields of the same patient during the follow-up (FU) period.

**Figure 3:**
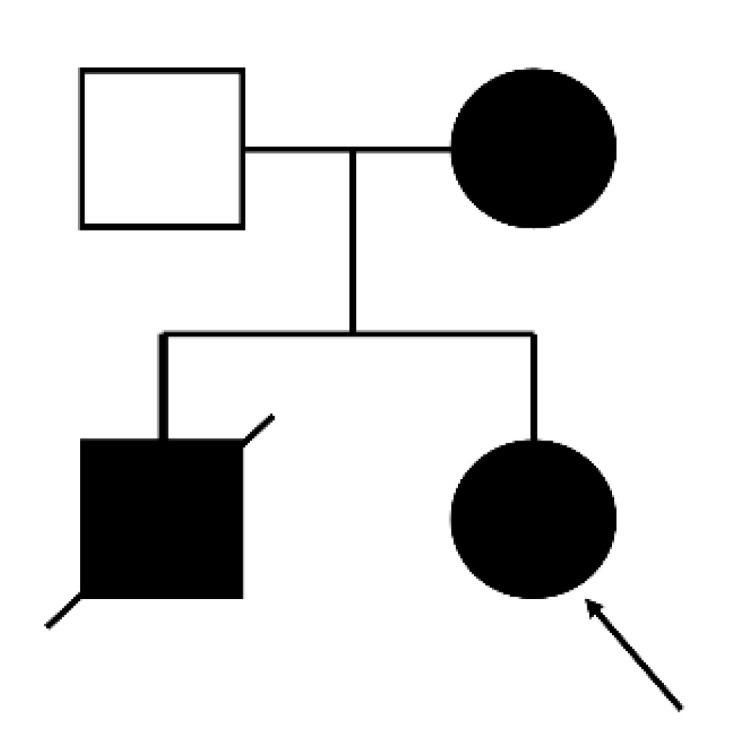
Pedigree of an Asian family with Danon disease. Arrow indicates the proband.

A 21-year-old Asian woman and her 51-year-old mother were referred to our clinic with suspected retinitis pigmentosa (RP). The patient’s brother had died in his mid-twenties due to acute rejection of a cardiac transplant received for hypertrophic cardiomyopathy, and her mother, father, and brother had arrhythmias. The patient had no visual symptoms and an unremarkable ophthalmic history. Her visual acuity was 20/20 in both eyes. Fundus examination revealed diffuse salt-and-pepper pigmentary changes in the peripheral retina, but optical coherence tomography (StratusOCT, Carl Zeiss Meditec, Dublin, CA, USA) of the macula revealed no abnormalities (Figure 1A,B). On fluorescein angiography, hypofluorescence consistent with pigmentary lesions was identified in the peripheral retina (Figure 1C). The patient’s mother had similar but milder peripheral lesions, which were hypopigmented. Atypical RP was suspected, and biannual follow-up evaluations were performed (Figure 2). During the follow-up period, no evident extension of peripheral pigmented lesions or visual field progression was observed (Humphrey field analyzer, Carl Zeiss Meditec, Dublin, CA, USA). Eleven years later, the patient developed atrial fibrillation with infiltrative myocardial fibrosis. Considering her young age, familial genetic testing was performed. A nonsense mutation in the *LAMP2* gene (c.123C>A; p.Cys41Ter) was identified in the patient and her mother, and Danon disease was diagnosed. Notably, the patient and her father had a duplication at c.386 of the *KCNE1* gene, causing a frameshift (p.Ter130MetfsTer7) (Figure 3). The Ethic Committee Approval number for an exemption is “2020-1544”.

**Figure 4:**
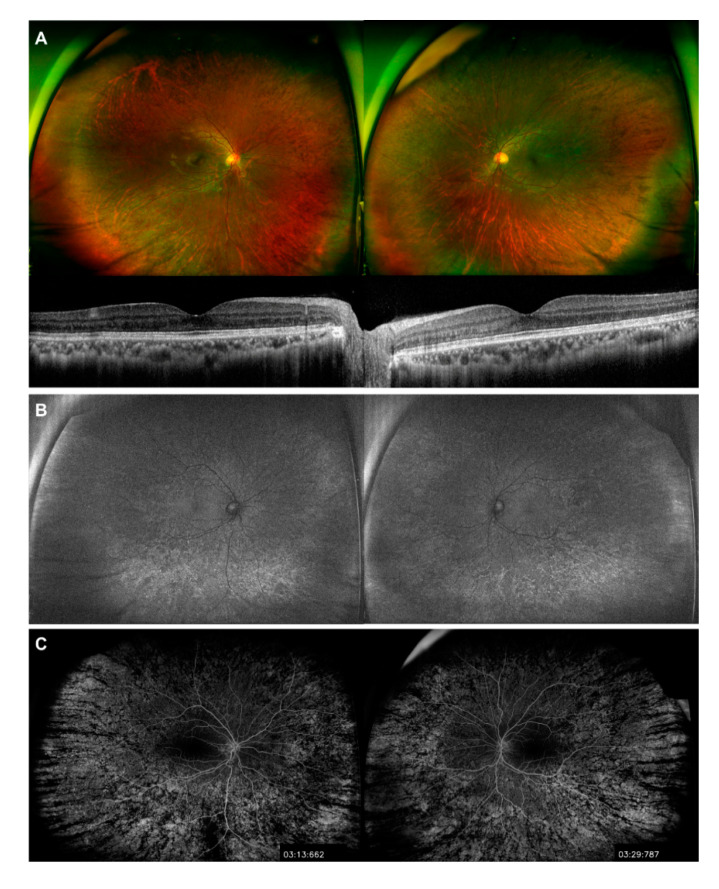
Images of peripheral retinopathy associated with Danon disease obtained after twelve years of follow-up (patient at 33 years old). (**A**) Ultra-widefield fundus images show an indolent peripheral pigmentary retinopathy that does not extend to the macular area (top). OCT images of both eyes show a well-preserved macular area (bottom); (**B**) ultra-widefield fundus autofluorescence; (**C**) fluorescein angiography images show diffuse retinal pigment epithelial atrophy confined to the peripheral retina without further extension to the macular area compared to the images of the initial visit.

**Figure 5:**
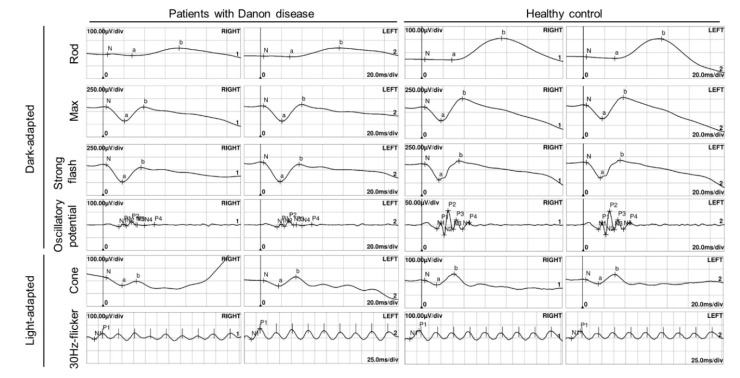
Electroretinogram of the patient after twelve years of follow-up (**left**) and healthy control (**right**).

**Figure 6:**
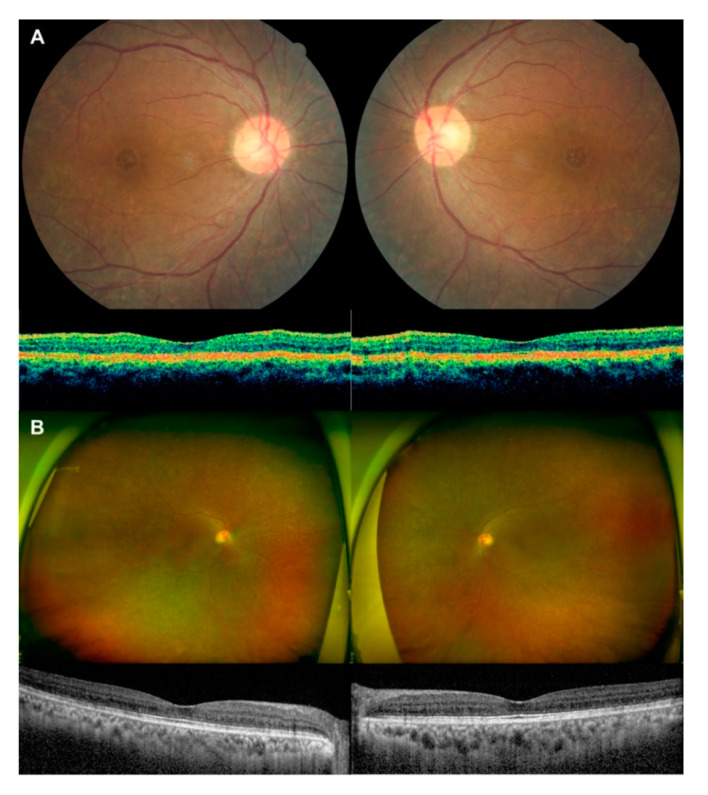
Images of the patient’s mother (a 63-year-old female): (**A**) fundus photograph and optical coherence tomography (OCT) at initial visit. Fundus photograph shows intact macular area (top), and OCT images showed unremarkable findings; (**B**) 12 years later, ultra-widefield fundus images showed hypopigmented peripheral lesions sparing the macular area (top), and OCT images revealed well-preserved outer retinal layers (bottom).

Twelve years after initial presentation, the patient showed no visual symptoms or deterioration in visual acuity. The pigmentary lesions were stable and had not extended further into the major vascular arcades (Figure 4A) (Wide field fundus photographs, Optomap, Optos PLC, Dunfermline, Scotland, UK; Spectralis OCT, Heidelberg Engineering, Heidelberg, Germany). Fundus autofluorescence revealed diffusely scattered areas of hypo- and hyper-autofluorescence corresponding to these lesions (Figure 4B). Fluorescein angiography revealed a pattern of hyperfluorescence and hypofluorescence consistent with window defects and hyperpigmentation in the peripheral retina, suggesting chorioretinal atrophy (Figure 4C). The lesions still spared the macula. The visual field was normal. Electroretinography (RETIport, Roland Consult, Brandenburg, Germany) showed a modest decrease in the rod response, which remained within the normal range (Figure 5). Comparably, her mother had shown a stable peripheral retinal lesion without further extension to the posterior pole compared to the fundus 10 years before (Figure 6).

## 3. Discussion

In this report, we have showed the longitudinal follow-up of peripheral pigmentary retinopathy in patients with Danon disease. The Danon disease-related retinopathy in an Asian patient featured an indolent macular-sparing peripheral lesion. Our cases showed that women have long-term preservation of macular integrity compared to men who have previously been reported to progress rapidly [3,4]. In addition, because peripheral retinopathy did not show significant progression in the patient and her mother, the rate of retinal disease progression may not have been affected by the patient’s age.

The site of *LAMP2* mutation in this patient (c.123 of exon 2) has not previously been reported [2]. Previous studies have reported two frameshift mutations (c.102–103, c179), and one nonsense mutation (c.138) in different positions in exon2 [5,6,7]. However, none of the patients reported a retinal disorder. It is unknown whether the specific mutation site in *LAMP2* is associated with the susceptibility to retinopathy [2,3,8,9]. Studies investigating the association between the mutation site in *LAMP2* and the retinal pathology could be valuable for suggesting retinal screening in specific subtypes of patients with Danon disease categorized by the mutation result.

Notably, the patient also had a *KCNE1* mutation; this gene encodes a potassium channel found in cardiomyocytes and retinal pigment epithelium (RPE) cells [10]. Although the microenvironment of the inner retina is strictly controlled by regulation of the tight junction and transcytosis of the inner blood–retinal barrier, homeostasis of the outer retinal microenvironment mainly depends on the RPE function [11,12,13,14]. *KCNE1* is localized in the basolateral surface of the RPE membrane and interacts with *KCNQ4* and *KCNQ5* channels to increase the potassium efflux and capacity of RPE to regulate the potassium current amplitude [10]. A function of the potassium channel is counter-regulation of the calcium influx [15]. Therefore, *KCNE1* mutation has the potential to disrupt the phagocytic function of RPE, which is affected by the potassium–calcium balance, [10,15,16] and potentiate the lysosomal dysfunction by *LAMP2* mutation. Since potassium homeostasis is important for maintaining RPE function, a lack of KCNE1 may aggravate phenotypes caused by LAMP2 deficiency. It remains to be elucidated whether the *KCNE1* mutation was responsible for retinal changes in this patient, or whether it was merely a bystander.

The indolent macula-sparing peripheral pigmentary lesions reported here are consistent with the relatively mild and stable macular phenotype of Danon disease previously described in female patients [3]. The *LAMP2* gene is essential for maintaining retinal homeostasis since LAMP2-deficient RPEs are susceptible to basal laminar deposits and subsequent atrophy [17]. The choriocapillaris has a role in removing basal laminar deposits and is responsible for facilitating nutrients and metabolites to the retina [18]. The choriocapillaris of the subfoveal area of the patients with Danon disease is relatively well-preserved, and LAMP2-deficient RPE of this region might have a better microenvironment to survive until an advanced age, thereby showing a “macular sparing” phenotype [19].

Interestingly, the patient’s mother, whose retinal lesions may reflect the likely progression of the patient’s lesions, retained well-preserved central vision and macular integrity into her 60s. The mother showed some minor annular hypopigmentary alterations in the macula that correspond to the hyper-reflective line at the inner limiting membrane level at the initial visit, but the outer retinal junctions in the OCTs remained intact. This abnormal finding may not be associated with LAMP2-deficient RPE pathology in Danon disease, which mainly involves changes in the outer retinal layer. However, careful monitoring of the macular pigment changes might be educational in providing a further understanding of the disease pathophysiology. The indolent and macular-sparing nature of the lesions may differentiate Danon disease from other pigmentary retinal dystrophies, such as RP, which involves progressive macular photoreceptor loss. Such prognostic information could be valuable for counseling patients anxious about losing their central vision. Though we report long-term macular sparing, maculopathy involving cone-rod dysfunction has been described in both male and female patients with Danon disease [4,9]. Differences in progression may be, in part, due to differences in ethnicity. Further long-term follow-up studies including patients from different ethnic backgrounds are necessary to clarify this issue.
